# Sporotrichosis: From KOH to Molecular Biology

**DOI:** 10.3390/jof4020062

**Published:** 2018-05-23

**Authors:** Roberto Arenas, Carlos D. Sánchez-Cardenas, Lourdes Ramirez-Hobak, Leon Felipe Ruíz Arriaga, Ma. Elisa Vega Memije

**Affiliations:** 1 Mycolgy Section, “Dr. Manuel Gea Gonzalez” General Hospital, Calz. de Tlalpan 4800, Belisario Domínguez Secc 16, Tlalpan, CDMX 14080, Mexico; rarenas98@hotmail.com (R.A.); lhobak@hotmail.com (L.R.-H.); leon.ruiz.a@gmail.com (L.F.R.A.); 2 Histopathology Department, “Dr. Manuel Gea Gonzalez” General Hospital, Calz. de Tlalpan 4800, Belisario Domínguez Secc 16, Tlalpan, CDMX 14080, Mexico; elisavega50@gmail.com

**Keywords:** sporotrichosis, *Sporothrix schenckii complex*, KOH, histopathology, molecular biology

## Abstract

Sporotrichosis is a cosmopolitan, chronic granulomatous mycosis, acquired by traumatic inoculation and caused by *Sporothrix schenckii complex*. Several methods of diagnostic are available, from KOH to molecular biology. In this review, we describe from the simplest (clinical diagnosis) to the most advanced diagnostic techniques (molecular biology).

## 1. Introduction

Sporotrichosis is a cosmopolitan, chronic granulomatous mycosis, acquired by traumatic inoculation and characterized by nodular lesions and caused by the dimorphic fungus *Sporothrix schenckii complex* [1].

Nowadays several methods of sporotrichosis diagnostic are available. In this review, we describe from the simplest (clinical diagnosis) to the most advanced diagnostic techniques (molecular biology).

## 2. Clinical Diagnosis

### 2.1. Acquisition, Transmission and Propagated of Disease

Sporotrichosis, known as “gardeners’ disease,” affects those especially involved in the cultivation of roses. Infection results from inoculation of the fungus by thorn, pricks, scratches, and other small injuries; a history of trauma can be absent. Certain occupational and leisure activities, such as floriculture, horticulture, gardening, fishing, hunting, farming, mining, and others that facilitate exposure to the fungus. In Uruguay and more recently in southern Brazil, the hunting of armadillos have been related to cases of sporotrichosis. In some situations, such as in an area of endemicity in Peru, the cat owners, outdoor activities, and low socioeconomic status were identified as risk factors for acquiring sporotrichosis. Some cases have been reported in laboratory professionals who were infected due the manipulation cultures of *S. schenckii*. Interhuman transmission is exceptional. Sporotrichosis usually occurs in isolated cases or small outbreaks in families and workers engaged in high-risk activities. Epidemics are rare and if they occur, are commonly related to a single source of infection [2].

### 2.2. Clinical Manifestations

Clinical features of sporotrichosis can be divided in cutaneous and extracutaneous disease, the former being the most common. Upper limbs are affected in 45–53%, face in 14–21% and lower limbs in 18–23% [2,3,4].

#### 2.2.1. Cutaneous Presentation

Cutaneous presentation has three variants: fixed, superficial form and cutaneous lymphangitic. Fixed presentation represents 20–30% of the cases. It is characterized by a painless, infiltrated, nodular lesion sometimes ulcerated or verrucous, with a violaceous halo in the site of inoculation (sporotrichoid chancre), instead of the superficial presentation, in which there’s lack of satellite lesions [2,3,4].

The lymphangitic form is the most common (80%) is characterized by ulcerated nodules in a linear distribution on the limbs [3,4,5]. Mucocutaneous variety has been described in conjunctiva, mouth, pharynx, vocal cords, nose, and sinuses. The lesions are granulomatous, ulcerous and painful. The simultaneous affection of the ocular mucosa and the regional lymph nodes is not rare, and it is one of the causes of Parinaud syndrome [6,7,8].

Immunoreactive presentations include erythema nodosum, erythema multiforme, Sweet’s syndrome and reactive arthritis [3,6].

Some patients show spontaneous regression. Without treatment patient could develop disseminated (extracutaneous) disease [3,6].

#### 2.2.2. Extracutaneous Presentation

Extracutaneous forms are uncommon and they involve in the majority of cases bones and joints causing periostitis, osteolysis and tenosynovitis as an extension of the disease. Pulmonary involvement could be acute or chronic. The first one is presented by cough, hemoptysis and consumptive symptoms on the other hand the chronic, is usually asymptomatic (98%) or indistinguishable from tuberculosis infection. Systemic sporotrichosis is considered a severe opportunistic infection and is observed mainly on immunosuppressed patients. It can affect almost every organ and could be lethal [3,4].

Central nervous system involvement is commonly due to *S. brasiliensis*; it occurs in about 17% of cases and causes signs and symptoms of meningoencephalitis and hydrocephalus [6,9,10].

In general, lesions leave fibrotic scars, causing alopecia depending of the affected area [11].

The differential diagnosis of cutaneous disease includes other infection and non-infection lesions that look alike verrucous tuberculosis, atypical mycobacterial infection, leprosy, mycetoma, chromoblastomycoses or squamous cell carcinoma. In case of pulmonary involvement differential diagnosis with tuberculosis is mandatory [3,7,11].

## 3. Direct Examination

*Sporothrix* fungal structures are difficult to observe in samples obtained from humans. Samples are obtained from the skin lesions (pus and exudates), sputum or synovial fluid. Potassium hydroxide (KOH) in a 10% to 40% dilution can be used for its visualization (this alkaline solution disrupt cellular sheets or clumps of proteinaceous debris that may be present, and clears the specimen for easier fungal detection), in which parasitic yeasts of 2–6 µm in diameter are observed, (1–2% of cases) [1,2].

Yeasts stain slightly positive to Gram staining and are commonly seen next to giant multinucleated or polymorphonuclear cells [3,10]. Periodic-acid Schiff (PAS) allows to see the elongated yeasts. Some authors have described the technique for Giemsa staining, in which the pus is diluted 10 to 15 times with physiological saline solution but it has very low sensitivity [3,10].

To observe the so-called asteroid bodies (yeasts surrounded by host immunoglobulins), also called Splendore–Hoeppli phenomenon, a technique is described in which the exudate of the lesions is placed on a slide with physiological saline solution and a drop of 10% formaldehyde solution, then observed at the microscope, this technique has a sensitivity of 93.75% and can be observed in 43% of cases. Due to its high sensitivity this technique allows an early diagnosis and treatment [4,12,13].

Currently direct immunofluorescence is performed in a few laboratories, a very sensitive diagnostic tool, but in underdeveloped countries, it is not a routine technique [14,15].

## 4. Fungal Culture

Isolation of the fungus on a culture is the gold standard for diagnosis. On Sabouraud dextrose agar at 28 °C, after 5 to 8 days characteristic colonies are formed, which are initially white, creamy, bright and yeast-like; later they turn white-beige, with dark pigment in the center, membranous, radiated and fast growing. At the microscopic examination, thin, septate, branched hyphae (1–2 µm in diameter) are observed with sympodulospores or radulospores, which gives the typical “daisy or peach flower” image [3,5,6]. The dimorphism of the fungus could be demonstrated by culturing it in blood-chocolate agar, blood agar or Brain Heart Infusion (BHI) agar at 37 °C [6]. Macroscopically creamy, yellowish, shiny colonies are observed, melanized conidia could be found at 25 °C [7,8].

## 5. Histopathological Study

**Figure 1:**
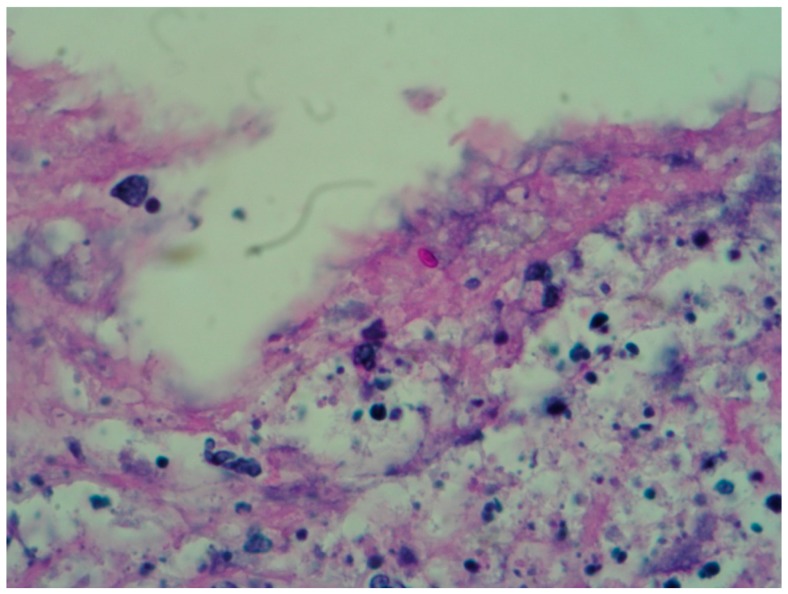
Skin biopsy stained with PAS in which yeast can be observed (40×).

**Figure 2:**
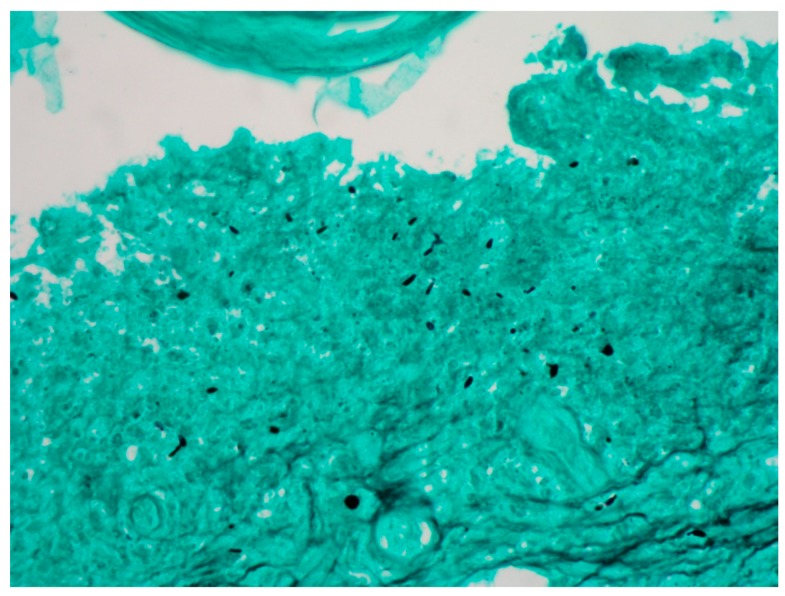
Grocott stain in which the cigar-shaped yeasts are observed (40×).

The most important use that is given to the tissue obtained from a biopsy is for the fungus culture because it is rarely identified in the histopathological study; so, two punch biopsies are taken (or only one and divided in two parts) to do both studies (culture and histopathology) [16]. Due to the difficulty to see the fungal structures in the tissue, a minimum of 20 serial sections to the paraffin block are recommended [17,18]. PAS and Gomori–Grocott stains are mandatory (Figure 1 and Figure 2) [17].

Typically, the epidermis can be observed with hyperkeratosis, parakeratosis, and pseudoepitheliomatous hyperplasia, as well as some polymorphonuclear microabscesse [1]. A suppurative or granulomatous reaction can be found in the dermis. According to Lurie and Still, histological patterns can be classified into sporotrichoid, tuberculoid or foreign body reactions [19]. Once the lesion has a long-term evolution, a granulomatous pattern is observed, with central necrotic microabscesses surrounded by epithelioid histiocytes, at the same time surrounded by plasma cells and lymphocytes; these are called the necrotic, tuberculoid and syphiloid zones respectively—this phenomenon its observed mainly in the nodules belonging to lymphocutaneous sporotrichosis [19,20]. There may be single or multiple granulomas, and the lesions do not have a capsule [20].

The histopathological characteristics of the fixed cutaneous sporotrichosis are: central ulceration of the epidermis, hyperkeratosis of the edges, acanthosis, epidermal hyperplasia; abscesses of neutrophils are observed in the dermis and epidermis. A dense inflammatory infiltrate is usually found, including lymphocytes, plasma cells, histiocytes, and eosinophils [20].

**Figure 3:**
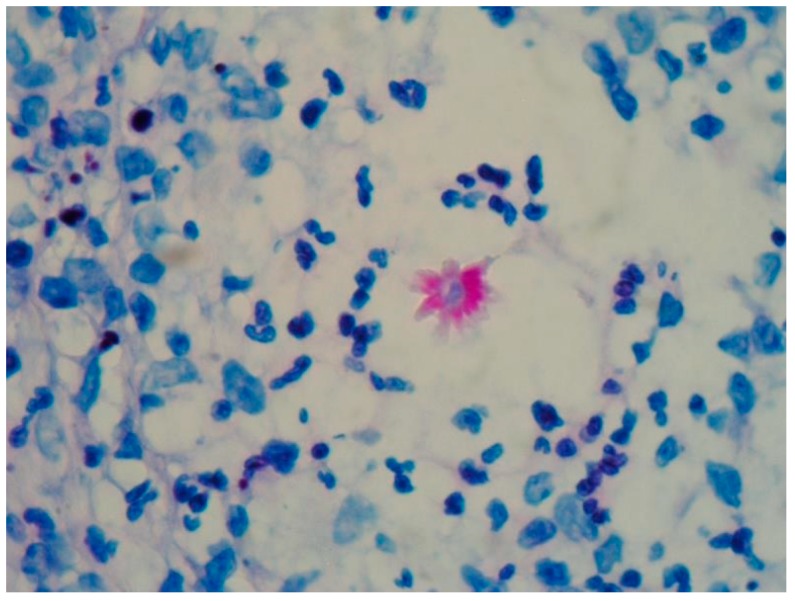
Sporotrichosis, an asteroid body surrounded by numerous neutrophils is observed (PAS, 40×).

**Figure 4:**
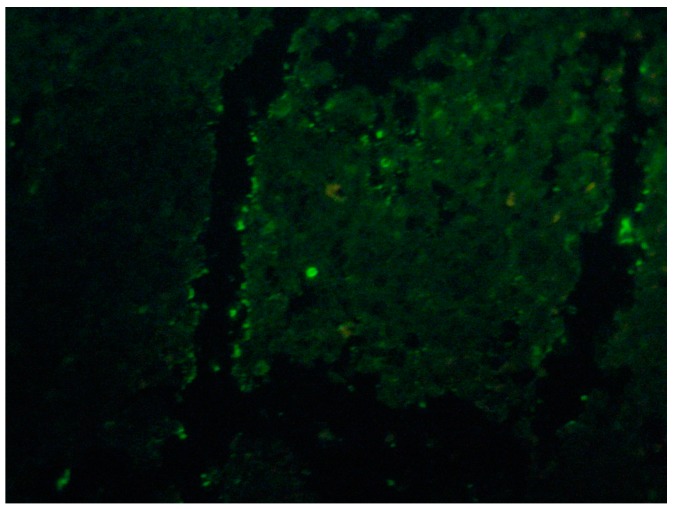
Skin Indirect immunofluorescence with sporotrichosis, fluorescent yeasts are observed (40×).

Some more specific staining for fungi such as Grocott’s metamine-silver (GMS) or periodic acid-Schiff (PAS) may reveal so-called “asteroid bodies” (Figure 3). These findings are not pathognomonic of sporotrichosis and can be observed in other granulomatous or infectious diseases like sarcoidosis and lobomycosis [10,16].The asteroid bodies from 15 to 35 µm in diameter, are found in 40 to 85% of the chronic cases inside the abscesses, mainly on fixed cutaneous sporotrichosis [16,21,22]. Asteroid bodies are better observed with PAS [20,21,22]. Its presence can be demonstrated in a more sensitive way by immunofluorescence or immunohistochemical techniques (Figure 4) [22,23].

Immunofluorescence trials have shown a sensitivity of up to 100% using rabbit immunoglobulins anti-spore and anti-*S. schenkii* yeast in human tissue biopsies with sporotrichosis [12].

The histological differential diagnosis includes diseases that present pseudoepitheliomatous hyperplasia and other granulomatous reactions, especially other fungal or mycobacteria infections. Deep mycoses, atypical mycobacteria, pyoderma type blastomycosis, halogenoderma, pyoderma gangrenosum, granulomatosis with polyangiitis (formerly Wegner’s granulomatosis) and other systemic vasculitis are also differential diagnosis [17,19].

## 6. Molecular Biology Diagnosis

Immunoenzymatic tests like ELISA and Western Blot are possible when antigens are characterized and standardized. They are useful for cutaneous, systemic and atypical presentations or when no clinical lesions are observed, it can also be applied to characterize the presence of the pathogen in clinical lessons. The antigen usually used is *Ss*CBF (*Sporothrix schenckii* Con A-Binding Fraction) that binds IgG antibodies and can be detected in skin, serum and other fluids with a sensitivity and specificity of 90% and 80% respectively. This test can be used to monitor treatment thanks to its high clinical correlation [6,22].

Cuomo et al. in 2014 sequenced the strain ATCC 58251 with a genome size of estimated 32.23 million bases (Mb) with a GC content of 55.2%. The same year the strain *Sporothrix schenckii* 1099-18 was also sequenced. Two years later the whole sequencing of *Sporothrix globosa* was described using CBS 120340 and SS01 strains finding the 34.51% of the entire genome [23,24,25]. Genomic analysis of *Sporothrix pallida*, a non-virulent strain, has shown a larger genome compared to pathogenic species [24,25].

There have been efforts to sequence more *Sporothrix* strains and other related organisms. As a result of this study it has been pointed out that *Ophiostoma novo-ulmi* and *Neurospora crassa* are closely related to the group [26].

PCR can be used to amplify specific genetic regions in order to identify a pathogen. That’s the case of ITS regions (Transcribed Spacer) that consist of two variable non-coding regions (ITS1 and ITS2) inserted between the highly conserved small subunit 18S, the 5.8S, and the large subunit 28S of the rDNA gene cluster. Estrada et al. used this method to identify *Sporothrix complex* from different geographic regions, however, this marker was unable to discriminate between species [6,23,27].

Other methods for species recognition are based on DNA sequences focused in genomic loci that encode proteins, such as calmodulin, beta-tubulin and translation elongation factor [23].

Sequence analysis of chitin sintetase genes, ß-tubuline and calmoduline has let us to identify *S. brasiliensis*, *S. schenckii*, *S. globosa*, *S. mexicana*, *S. albicans* and *S. luriei.* It has been documented an epidemic zoonosis afecting domestic cats (*Felis catus*) caused by *S. brasiliensis* in Brazil [27].

A study of conserved ribosomal region (ITS) and non-transcribed space (NTS) showed that *S. schenckii sensu strictu* of disseminated forms present a 10 bp deletion in NTS, comparing with fixed forms [28].

Phylogenetic analysis based on the rDNA and b-tubulin regions from *Sporothrix albicans*, *Sporothrix pallida* and *Sporothrix nivea* revealed significant similarity, with the proposal of designating all of these species as *S. pallida* [6,24].

Analysis of phylogeny and genetic diversity using calmodulin DNA sequences showed the phylogenetic proximity between *S. schenckii s. str*. and *S. brasiliensis*, *S. globosa*, *S. mexicana* and *S. pallida*, sequences showed highly differentiated species. Phylogenetic tree suggests that *S. mexicana* shared a common ancestor with *S. pallida*; while *S. globosa*-*S. brasiliensis* are more related to *S. schenckii s. str*. and showed less haplotype diversity and restrictions in geographic distribution [25].

**Figure 5:**
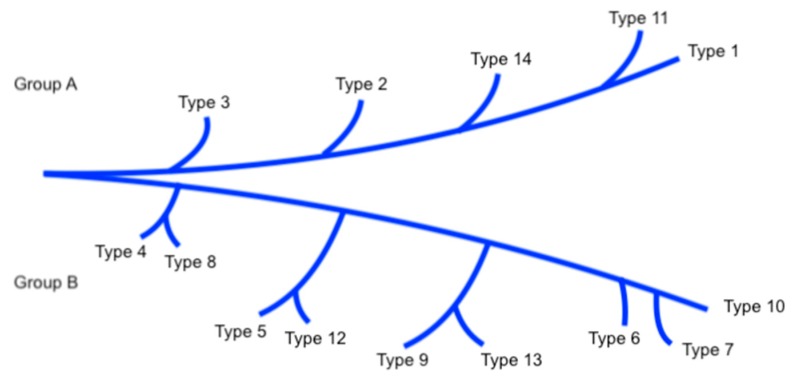
Phylogenetic tree of *Sporothrix schenckii*.

With PCR-RFLP (restriction fragment lenght polymorphism) it is possible to identify species of medical interest: *S. brasiliensis*, *S. schenckii s. str.*, *S. globosa*, and *S. luriei*. The environmental species *S. pallida* and *S. mexicana* had identical profiles that were distinct from those observed in the isolated clinical species in the *S. schenckii complex*. Distinct profiles produced by analysis of different species by this method can be classified in groups A and B. Species in each type are closely related to each other and show a geographical aggregation. American isolates belong to group A and those from Japan belong to group B. Remarkably, type 14 has been found in a high frequency in Latin America [3,6,26] (Figure 5).

The nuclear marker CAL obtain by PCR is consider the gold standard in molecular methods because it clearly separate species between clades in the phylogenetic tree. RFLP of the CAL locus allows rapid, species-specific identification of *S. schenckii complex*. The method is particularly useful when large numbers of strains have to be processed in case of an epidemic [29,30].

Nested PCR can help identify species even in difficult conditions [31].

Thanks to the PCR techniques, sporotrichosis has been related with the theory of evolution, because types 3 and 4 (mtDNA) shows a worldwide distribution. Japanese researchers think that ancestral organisms derived in the course of continental separation and this divergence in South Africa occurred before than in Australia [32].

Rolling Circle Amplification (RCA) is capable of synthesizing large DNA amounts based on very low initial concentrations [6].

It can also identify species by matrix-assisted laser desorption ionization time-of-flight mass spectrometry (MALDI-TOF MS). It generates a spectrum that is characteristic of each fungal taxon based on their proteomic profiles. In a study performed by Evangelista et al. seventy clinical and environmental *Sporothrix* isolates were identified using the standard protocol for yeast. They compare the results with DNA sequencing of the CAL gene with, a confidence interval superior to 99% [33,34,35,36].

Susceptibility profiles of species with medical importance (*Sporothrix brasiliensis*, *Sporothrix schenckii sensu lato*, *Sporothrix globosa*, and *Sporothrix luriei*) showed that itraconazole and posaconazole are moderately effective against *S. brasiliensis* and *S. schenkcii*. Posaconazole presents good minimal inhibition concentrations against *S. mexicana*. Fluocytocine, caspofungine and fluconazole showed no fungal activity against *Sporothrix* species. These results highlight the importance of rapid molecular identification of etiologic agents in order to inform therapy of choice [24].

These methods are important because of their high sensitivity and specificity to identified the sporotrichosis species and know the different susceptibility to treatment of every one of them, this is an advantage then the classical methods of diagnostic.

This type of diagnosis would provide in the future opportunities to highlight the understanding of *Sporothrix* species. Using comparative genomics for localize regions or factors that determine the variability of virulence, resistance to treatment or other characteristics would improve therapeutics and better outcome for patients. Phylogenetic trees will facilitate the study of *Sporothrix schenckii* group and clarify the taxonomy of the species in this genera.

## 7. Immunological Tests

Skin test has been used as an intradermal reaction since 1947 by González-Ochoa in Mexico [15,37]. In the United States and some European Union countries its use has not been accepted because the process it’s not standardized. In Mexico, is considered a useful tool because it informs us about the immune status of a patient, mainly the cellular immunity, as well as to carry out epidemiological works [22,38,39,40]. It helps us to know the level of hypersensitivity against the fungus [41,42]. The immunological test is useful in both, cutaneous and extracutaneous sporotrichosis.

Currently, it is obtained from chemically defined culture media, which are then dialyzed to increase its efficiency and reduce cross-reactions [41,43].

*S. schenckii* is a dimorphic fungus, so its antigens are divided as mycelial (M) and yeasts (L); both are formed by glycopeptides and polysaccharides with mannose, sucrose, glucose and l-rhamnose. l-rhamnose conforms the cells walls of very few pathogenic fungi, which makes it a relatively specific antigen [13,43,44]. The mycelial antigenic polysaccharide fraction is diramnosyl-framnomannans, and the yeast-like is constituted by monoramnosyl-rhamnomannans [3].

Technique: 0.1 mL of the antigen solution is injected and lecture 48 h later; it is considered a positive test when a lesion measuring ≥5 mm is formed [45,46]. The test can be false negative in patients with cellular immunity alterations, mainly AIDS, diabetes mellitus, and in patients with disseminated sporotrichosis [47].

It has been reported that the antigen cross-reacts with *H. capsulatum* [45]. Saul and Bonifaz described a classification of the disease according to the immunological reaction to the skin test [44] (Table 1).

## 8. Conclusions

In conclusion, there are different methods to confirm the diagnosis of sporotrichosis. Clinical diagnosis is important to exclude other infections and identify the different forms of sporotrichosis. Subsequent studies are necessary to confirm the diagnosis, either directly under the microscope by applying potassium hydroxide on purulent exudate from the lesion or the histopathological study. Isolation on Sabouraud dextrose agar is mandatory for phenotypical identification of the etiological agent. Currently, there are molecular biology techniques available as well as immunoassay, mass spectrometry, among others for the precise identification and confirmation of the causal fungi.

## Figures and Tables

**Table 1:** Sporotrichosis classification.

Inmunology and Clinical Parameters	Group I	Group II
Immune Response	Normal/Augmented	Decreased/None
**Clinical presentations**	Lymphangitic Fixed	Cutaneous disseminated Cutaneous superficial Pulmonary/visceral Osteoarticular
**Parameters**	Skin test positive (+) Scarce yeasts or asteroid bodies.	Skin test negative (−) Plenty of yeasts or asteroid bodies.
